# Oral Contraceptive Pills Are an Effective Method of Preventing Pregnancy in Women With Crohn’s Disease

**DOI:** 10.1093/crocol/otab078

**Published:** 2021-11-13

**Authors:** Nader D Daoud, Hassan Ghoz, Rachel Cannon, Jennifer A Farraye, Michael F Picco, Sunanda V Kane, Gursimran S Kochhar, Elisabeth J Woodhams, Francis A Farraye

**Affiliations:** 1 Division of Gastroenterology and Hepatology, Mayo Clinic, Jacksonville, Florida, USA; 2 Department of Obstetrics and Gynecology, Boston Medical Center, Boston, Massachusetts, USA; 3 Division of Gastroenterology and Hepatology, Mayo Clinic, Rochester, Minnesota, USA; 4 Department of Gastroenterology, Hepatology and Nutrition, Allegheny Health Network, Pittsburgh, Pennsylvania, USA

**Keywords:** oral contraceptive pill efficacy, Crohn’s disease, malabsorption

## Abstract

**Background:**

Oral contraceptive pill (OCP) use in the general population is associated with a failure rate as low as 0.3% with perfect use but as high as 9% with typical use. Women with Crohn’s disease (CD) may have malabsorption in the setting of small bowel disease or resection, which could affect absorption of OCPs. Our aim was to determine the incidence of pregnancy in women with CD on OCPs.

**Methods:**

This is a retrospective study assessing the incidence rate of OCP failure in females between 18 and 45 years of age seen at the Mayo Clinic with a diagnosis of CD and provided a prescription for OCPs, between 2016 and 2020. Failure was defined as clear documentation of becoming pregnant while using OCPs or having an active prescription of OCP at the time of conception.

**Results:**

A total of 818 female patients with CD between 18 and 45 years of age with a prescription for an OCP were included in our study. Sixty-six patients (8%) conceived in this cohort. Of the 66 patients who became pregnant, 57 stopped the OCP before conceiving, 5 were excluded due to lack of data, and 4 women had active oral contraceptive prescriptions when they became pregnant (pregnancy rate of 0.5%).

**Conclusions:**

In female patients with CD who are using OCPs for contraception, we found a low rate of pregnancy (0.5%) similar to the rate of pregnancy with perfect use of OCPs in the general population. OCPs are an effective method of birth control in women with CD.

## Introduction

Oral contraceptive pills (OCPs) are a popular contraceptive method, used by more than 100 million women worldwide.^1^ There are 2 main types of OCPs: progestin-only pills and combined hormonal pills with the latter being the most frequently prescribed.^2, 3^ Failure rates vary significantly, with 0.3% failure rate attributed to “perfect use”^2^ and 9% failure rate attributed to “typical” use.^3^ Failure of OCPs may lead to unintended or unplanned pregnancy which can be associated with adverse maternal and neonatal outcomes.^4, 5^

The incidence of Crohn’s disease (CD) and ulcerative colitis is increasing and affects hundreds of thousands of women in the United States.^5^ The diagnosis of inflammatory bowel disease (IBD) peaks during a woman’s reproductive years, emphasizing the importance of contraception and pregnancy counseling by health care providers.^5^ Multiple studies have analyzed different aspects of pregnancy on CD activity and the impact of CD on pregnancy outcomes.^6^ Disease activity was more likely to remain active in women who became pregnant during a CD flare. In addition, there is an increase in adverse pregnancy outcomes in women with ongoing disease activity during pregnancy.^7^ In contrast, women who became pregnant while their CD was inactive are more likely to remain in remission and have favorable pregnancy outcomes.^8–10^ Educating women with CD and their providers on avoiding conception during disease activity and continuing maintenance therapy during pregnancy is crucial.

Micronutrient malabsorption and the chronic inflammatory state can play a causative role in CD adverse pregnancy-related outcomes.^11^ Chronic inflammation of the intestinal mucosa, intestinal surgical resection, or intestinal obstruction cause a state of chronic malabsorption in women with CD.^12^ Micronutrient deficiency is common in CD where iron deficiency, among other vitamins and minerals, is the most common.^13^ Since most oral contraceptive steroids are absorbed in the small intestine, intestinal inflammation, ulceration, or surgical resection associated with CD can potentially affect the absorption and bioavailability of OCPs.^14–16^

Limited data consider the efficacy of OCPs in women with CD. In a prospective study of the pharmacokinetics of high-dose OCPs in women with IBD undergoing proctocolectomy and construction of a continent ileostomy, absorption of the OCPs was not different from that in women without IBD.^17^

Given the possibility of malabsorption associated with small bowel disease or resection in women with CD, patients and physicians often question the efficacy of contraceptive pills to prevent unintended pregnancies with its numerous complications. We hypothesize that the rate of OCP failure is not different in women with CD on OCPs when compared to women without CD. We designed a retrospective study to assess the incidence of pregnancies in women with CD who are on OCPs, analyze CD disease characteristics and the associated patient demographics.

## Methods

### Study design and patient population

This was a retrospective study performed at Mayo Clinic sites in Jacksonville, Florida, Phoenix, Arizona, and Rochester, Minnesota. A search of the Mayo Clinic electronic medical record (EMR) was used to identify cases and demographic factors. Within the EMR, a clinical data repository contains patient demographics, ICD-9 and ICD-10 coded diagnoses, laboratory data, clinical and operative notes, and medications with numerous search options. We used Epic’s recently implemented SlicerDicer search engine, which dictated our inclusion time frame. This search engine allowed us to capture a category of medications (“contraception”) regardless of the generic or brand name and filter by route of administration (oral). It allows identification of patients who became pregnant using ICD-10 codes, labs, and or documentation. Between January 1, 2016 and November 23, 2020, we searched the EMR for females with ICD-9 or ICD-10 code for CD, aged 18 through 45, with a prescription for an OCP (either combined hormonal contraceptive pills or progesterone-only contraceptive pills) who became pregnant. After patients were identified, a manual chart review was performed to extract additional clinical characteristics and outcomes. We used medication history and clinical documentation to confirm OCPs use. We used ICD codes and clinical documentation to confirm the presence of CD, obtain information about the presence or absence of psychiatric disorders, nulliparity, or multiparity status. Other collected data included age, race, insurance status, insurance type, body mass index (BMI), and zip code. The official website for United States Census Bureau, census.gov, was used to obtain the percentage of females under the poverty line by zip code. Clear documentation of discontinuing OCPs to attempt pregnancy was used to rule out OCPs failure even in the presence of an active OCPs prescription. Either clear documentation of pregnancy in the setting of typical use of OCPs or active OCPs prescription during conception was used to diagnose pregnancy while utilizing OCPs. This study was approved by the Mayo Clinic Institutional Review Board.

### Statistical analysis

Descriptive statistics for continuous variables such as subject characteristics and basic demographics were reported as sample median and interquartile range in view of their parametric distribution. Categorical variables were summarized with the number and percentage of patients. Statistical analysis was performed using STATA.

## Results

In total, 3969 women had CD, of which 818 met the criteria for study inclusion. Sixty-six patients (8%) conceived in this cohort. Out of the 66 who became pregnant, 57 stopped the OCP before conceiving, 5 were excluded due to lack of data, and 4 women had active oral contraceptive prescriptions when they became pregnant with a pregnancy rate of 0.5%.

Women who became pregnant while utilizing OCPs (*n* = 4) had a median age of 24.5 years, were White (100%), had a median BMI of 21.5, were insured (100%), had a median of 9.6 % of females below the poverty line, 3 out of 4 (75%) were nulliparous, and 2 (50%) had an associated anxiety and depression diagnosis.

Women who became pregnant after discontinuing the OCP (*n* = 57) had a median age of 32 years, were White (100%), had a median BMI of 25, were insured (100%), had an average of 10.6% of females below the poverty line, 29 (51%) were nulliparous, 21 (36.8%) had an associated anxiety disorder, and 25 (43.8%) had an associated depression disorder. Table 1 summarizes descriptive statistics of both groups.

**Table 1.** Summary of patient demographics.

	Pregnancy in women on OCPs (4)	Pregnancy in women off OCPs (57)
Age (years, median IQR)	24.5 (22.5–34)	32 (27–34)
BMI (median, IQR)	21.5 (19.5–22.5)	25 (22–29)
% Below poverty line (median, IQR)	9.6 (7.45–11.8)	10.6% (7.8–14.8)
Anxiety (*N*, %)	2 (50%)	21 (36.8%)
Depression (*N*, %)	2 (50%)	25 (43.8%)
Nulliparous (*N*, %)	3 (75%)	29 (51%)

Abbreviations: OCP, oral contraceptive pill; IQR, interquartile range; BMI, body mass index.

Of the 4 OCP users who became pregnant, 1 had documentation of conception while taking a progestin-only pill. The other 3 patients were prescribed a combined hormonal contraceptive pill (ethinylestradiol and progestin) at the time of conception. On further analysis of the 4 patients with pregnancy, 3 were in their 20s and 1 was 42 years old. BMI ranged from 18 to 24. Two had ileocolonic (L3) involvement and 2 had colonic (L2) involvement. Colonoscopy performed prior to the time of pregnancy showed that 3 had moderate to severe inflammation and 1 had a normal colonoscopy as noted in Table 2. One patient had a flare around conception, 1 had a flare in her third trimester, and the other 2 were in clinical remission around the time of conception and throughout their pregnancy. None of the 4 women had a prior history of small bowel or colonic resection, diagnosis of celiac disease, or vitamin B12 deficiency.

**Table 2.** Patient characteristics of women who became pregnant while on OCPs.

Patients/Characteristics	Patient A	Patient B	Patient C	Patient D
Age	22	23	26	42
BMI	24	21	23	18
OCP type	Combined hormonal contraception	Combined hormonal contraception	Combined hormonal contraception	Progestin-only pills
Extent of CD	Colonic (L2)	Ileocolonic (L3)	Ileocolonic (L3)	Colonic (L2)
Colonoscopy ­findings	Severe colonic ­inflammation	Severe ileal and colon inflammation	Normal colonoscopy	Moderate colonic inflammation
Disease flare	Around conception	Third trimester	None	None
Previous surgical resection	None	None	None	None
CD medications	Adalimumab and prednisone	Vedolizumab	Infliximab	Tofacitinib
Vitamin B12 deficiency	None	None	None	None

Abbreviations: OCP, oral contraceptive pill; CD, Crohn’s disease; BMI, body mass index.

## Discussion

OCP use in the general population is associated with a failure rate as low as 0.3% with perfect use, but as high as 9% with typical use.^2, 3^ CD can be associated with nutrient malabsorption in the setting of small bowel disease or resection, which could affect absorption of OCPs.^14–16^ Our aim was to determine the incidence of pregnancy in women with CD on OCPs and further analyzing this subset of patients for likely risk factors and predictors of failure.

Our data showed that the incidence rate of OCP failure in women with CD (0.5%) is similar to the incidence rate of pregnancy in the general population (0.3%) with perfect use. Women in both groups were White (100%) and insured (100%), likely due to sample bias given the racial and socioeconomic demographics of our clinical sites, respectively. The national average for women below the poverty line was 13.6% in 2017 and 12.9% in 2018. Women in both groups had a lower percentage of females below the poverty line, with women who became pregnant off OCPs at 10.6%, and 9.6% in women with pregnancy on OCPs, likely a sample bias explained by the geographic location of our clinical sites. Women who became pregnant while utilizing OCPs were younger, more likely to be nulliparous, have lower BMI and lower percentage below the poverty line, and more likely to have anxiety and depression, than women who became pregnant after discontinuing OCPs.

Combined hormonal pills are associated with lower failure rates than progesterone-only pills; however, we found that women who became pregnant while on OCPs, 3 (75%) were on combined hormonal contraception and 1 (25%) were on progestin-only pills.^1, 18^ This may have been due to very small sample size. Patients with CD can develop malabsorption secondary to chronic intestinal inflammation or resection.^11, 12^ Although numbers were small, our data showed that 3 (75%) of the women who became pregnant while on OCPs group had moderate to severe inflammation on a colonoscopy performed prior to conception; however, 2 (50%) had only colonic disease and none had intestinal or colonic resection.

Perhaps surprisingly, our data may suggest that women with CD may have less risk of OCP failure than the general population, given the low rate of pregnancy which more closely approximates perfect-use failure rates than typical-use failure rates. The distinction between typical-use and perfect-use failure rates of OCPs in the general population reflects in part real-world challenges in maintaining a daily routine of utilizing a medication.^19^ Patients with chronic disease such as CD, however, may be more accustomed to a daily medication and prefer something that integrates into their routine. These patients may be more motivated to time a pregnancy to avoid worsening of chronic disease or may be health conscious in general.^20–22^ Future studies should consider incorporating patient perspectives and experiences with varying contraception methods to better understand this possible finding.

Our study had several limitations. All participants included in the study were White and had medical insurance. We recognize that the study population does not reflect the national population of patients with CD. In view of the retrospective nature of the study, we used active OCP prescriptions to confirm if the patient was taking her OCP. We are aware that it is possible that some patients could be taking OCP prescribed by their primary care doctor and not listed in their medications at Mayo Clinic, which if properly documented could potentially have led to a larger sample size. We identified only 66 women with CD who had an OCP prescription and became pregnant. We did not have a matched control group of women without CD on OCPs to determine the rates of pregnancy while using OCPs. We did not have detailed information about the OCPs previously used in the women who became pregnant after discontinuing the OCP group nor could we assess adherence with OCP in the women who became pregnant while utilizing OCPs.

Our finding of similar rates of pregnancy for women taking OCPs compared to the general population is generalizable to a cohort of insured Caucasian women with a low rate of poverty but may not apply to all women with IBD. Our observations regarding type of OCP, relationship to disease activity, and distribution and pregnancy are important in that they should lead to further study. Since limited data are available on the efficacy of OCPs in CD, future studies should consider involving multiple sites in different geographic areas to increase the sample size and diversity of the women studied. Prospective studies can focus on questionnaires assessing patients’ compliance with OCP on daily basis, disease activity to assess disease flare or remission status, endoscopic evaluation especially if the patient is planning to become pregnant, detailed current and past CD medications, and pregnancy-related maternal and neonatal adverse outcomes.

In summary, we found that female patients with CD who are using OCPs for contraception have a low rate of pregnancy (0.5%) similar to the rate of pregnancy with perfect use of OCPs in the general population (0.3%).

## Data Availability

The data underlying this article will be shared on reasonable request to the corresponding author.
